# Ultra-High Dose Rate (FLASH) Radiotherapy: Silver Bullet or Fool's Gold?

**DOI:** 10.3389/fonc.2019.01563

**Published:** 2020-01-17

**Authors:** Joseph D. Wilson, Ester M. Hammond, Geoff S. Higgins, Kristoffer Petersson

**Affiliations:** ^1^Department of Oncology, The Oxford Institute for Radiation Oncology, University of Oxford, Oxford, United Kingdom; ^2^Radiation Physics, Department of Haematology, Oncology and Radiation Physics, Skåne University Hospital, Lund, Sweden

**Keywords:** FLASH, radiotherapy, hypoxia, normal tissue, immune

## Abstract

Radiotherapy is a cornerstone of both curative and palliative cancer care. However, radiotherapy is severely limited by radiation-induced toxicities. If these toxicities could be reduced, a greater dose of radiation could be given therefore facilitating a better tumor response. Initial pre-clinical studies have shown that irradiation at dose rates far exceeding those currently used in clinical contexts reduce radiation-induced toxicities whilst maintaining an equivalent tumor response. This is known as the FLASH effect. To date, a single patient has been subjected to FLASH radiotherapy for the treatment of subcutaneous T-cell lymphoma resulting in complete response and minimal toxicities. The mechanism responsible for reduced tissue toxicity following FLASH radiotherapy is yet to be elucidated, but the most prominent hypothesis so far proposed is that acute oxygen depletion occurs within the irradiated tissue. This review examines the tissue response to FLASH radiotherapy, critically evaluates the evidence supporting hypotheses surrounding the biological basis of the FLASH effect, and considers the potential for FLASH radiotherapy to be translated into clinical contexts.

## Introduction

In the UK, almost 30% of diagnosed tumors are treated with radiotherapy (RT) (1). External beam RT is a non-invasive procedure whereby tumors are targeted with ionizing radiation causing lethal damage to cancer cells resulting in cell death. However, RT also inflicts acute and chronic toxicities to the normal tissue surrounding the tumor (2–6). These radiation-induced toxicities limit the dose of radiation that can be delivered and subsequently limits the extent to which RT can be curative. Furthermore, as the number of long-term cancer survivors increases, late onset toxicities resulting from RT are emerging that significantly impact the quality of life of those patients. Consequently, there is a need for novel RT strategies that maintain the anti-tumor effect whilst limiting the extent of toxicities induced in the surrounding healthy tissue. Limiting the induction of toxicities to normal tissue would subsequently increase the therapeutic index of RT regimes (7). A number of recent studies have demonstrated that irradiation at ultra-high dose rates (FLASH) diminishes the severity of toxicities in normal tissues compared to irradiation at the conventional dose rates (CONV) currently used in clinical practice (8–18). Notably, limited data also shows that FLASH-RT reduces normal tissue toxicities whilst maintaining the anti-tumor response of CONV-RT (8–10, 15, 17, 19). FLASH-RT delivery uses irradiators with a high radiation output that allows for the entire RT treatment, or large fraction doses, to be delivered in parts of a second, compared to several minutes for CONV-RT. The short treatment times used in FLASH-RT, often shorter than 0.1 s, have the added value of minimizing treatment delivery uncertainties caused by intra-fraction motion. Carefully implemented, this would allow for smaller treatment margins and therefore smaller volumes of normal tissue being unnecessarily irradiated. Given both the radiobiological advantageous FLASH effect and its potential to “freeze” physiological motion (15, 20), FLASH-RT has the potential to be an important evolutionary step in cancer treatment. The biology underpinning the FLASH effect, however, remains unknown.

## Flash-Rt Limits Normal Tissue Toxicity

Investigation of the dose rate at which RT is delivered harks back to the 1960s, when it was demonstrated that non-cancerous mammalian cells irradiated at ultra-high dose rates had greater viability than those irradiated at conventional dose rates (21). More recently, this toxicity-limiting property of ultra-high dose rate was rediscovered and named FLASH by Favaudon et al. (10). In their study, they demonstrated that thoracic irradiation of mice with a single fraction of 17 Gy at conventional dose rates (0.03 Gy/s) induced “moderate” and “severe” regions of pulmonary fibrosis at 36 weeks post-irradiation. In contrast, when mice received the same dose at ultra-high dose rates (40–60 Gy/s) the induction of pulmonary fibrosis was starkly reduced. A greater dose of 30 Gy delivered by FLASH-RT was required to induce comparable levels of pulmonary fibrosis as seen following CONV-RT (10). Whilst exploring this reduction in pulmonary fibrosis following FLASH-RT, the same group investigated any changes in the induction of the transforming growth factor beta (TGFβ) signaling cascade—a well-documented molecular marker of radiation-induced pulmonary fibrosis (22). In accordance with their prior findings, CONV-RT of 17 Gy significantly induced TGFβ signaling; this signaling was reduced in mice that had been subjected to FLASH-RT. Once again, a greater dose of 30 Gy delivered by FLASH-RT was required to induce TGFβ signaling to the equivalent extent as seen following irradiation with CONV-RT (10). Limited TGFβ signaling following FLASH-RT has also been shown *in vitro* (23): this study demonstrated that even 24 h post-irradiation, CONV-RT induced 3-fold greater TGFβ signaling compared to FLASH-RT.

In addition to thoracic irradiation, it has been shown in several studies that whole brain irradiation using FLASH-RT confers neuroprotection compared to CONV-RT (13, 14, 24, 25). In one such study, mice were exposed to varying dose rates, ranging from 0.1 Gy/s to 10 Gy delivered in a single 1.8 μs pulse; at all dose rates mice were exposed to 10 Gy in a single fraction (14). Any radiation-induced neurotoxicity was measured by a novel object recognition test 2 months post-irradiation. Analysis of these data showed that mice irradiated at 0.1 Gy/s performed significantly worse on the novel object recognition test compared to the non-irradiated control. Notably, as dose rate increased, mice performed significantly better in the recognition test when irradiated at dose rates ≥ 30 Gy/s. Furthermore, there was no statistical difference in novel object recognition between mice irradiated at dose rates exceeding 100 Gy/s and non-irradiated mice (14).

In earlier studies, it was observed in rodent models that radiation-induced skin reactions could be significantly reduced at ultra-high dose rates (26, 27). Specifically, it was shown in a rat model that irradiation at 67 Gy/s induced less severe skin reactions, e.g., reddening, moist desquamation, and skin breakdown, in the short and long term compared to rats irradiated at either 1 or 0.03 Gy/s. This study also measured the deformity of the irradiated feet 6 months post-irradiation; consistent with the induction of skin reactions, the extent of deformation was less in the rats irradiated at 67 Gy/s compared to the two lower dose rates (26). Pre-clinical FLASH-RT studies have also been extended from rodent models to higher mammals such as mini-pigs and cats (16). As recently and succinctly reviewed (28), this study irradiated ten 26 mm in diameter circular patches of skin on the back of a single mini-pig to five different dose levels from 22 to 34 Gy (in 3 Gy increments), with either FLASH-RT at a dose rate of 300 Gy/s, or CONV-RT at 0.083 Gy/s. Examination 48 weeks post-irradiation showed that FLASH-RT had been well-tolerated, with only mild cutaneous depigmentation at the site of irradiation (16). In contrast, sites subjected to CONV-RT presented with clear fibronecrotic lesions. By way of extension, this study used FLASH-RT to treat six cats, all presenting with squamous cell carcinoma of the nasal planum, to a total dose ranging from 25 to 41 Gy. All six cats responded extremely well to treatment with complete remission of tumors with minimal toxicity; cats treated with the largest doses of radiation exhibited moist desquamation around the site of irradiation (16). An obvious limitation of this study is the lack of a parallel arm of cat subjects treated with CONV-RT.

Many pre-clinical studies have reported a successful FLASH normal tissue sparing effect, but it cannot be overlooked that there have also been several studies reporting no significant sparing of normal tissues following irradiation at ultra-high dose rates (29–33). For example, Smyth et al. delivered whole and partial body (abdominal or head) synchrotron irradiation to mice, at ultra-high dose rates of 37–41 Gy/s in the hope of characterizing the equivalent CONV-RT dose (32). However, comparing TD_50_ values (dose predicted to cause toxicity, i.e., >15–20% weight loss, severe diarrhea, moribund behavior, in 50% of the animals), this study did not observe any differential sparing between broad beam irradiation of ultra-high and conventional dose rates. A similar study by Montay-Gruel et al. delivering whole brain synchrotron irradiation at a dose rate of 37 Gy/s to mice, did however show significant neurocognitive sparing compared to conventional X-ray irradiation (24). Synchrotron irradiation beams are very flat, several cm in width but with a height on the μm-mm scale, requiring the irradiated sample to be scanned through this beam slice. For studies investigating the FLASH effect with synchrotron irradiation, the dose rate within the beam slice is likely the most important parameter. So even though the average dose rate was similar in these two studies, and probably just high enough for a FLASH sparing effect (14), the height of the beam slice through which the mice were scanned was different by a factor 20 (50 μm compared to 1 mm), corresponding to the same difference in dose rate in the slice (12 000 Gy/s compared to 600 Gy/s) (14, 32). This difference in beam slice dose rate, and of course the difference in the investigated end-points, could explain why one study found a FLASH sparing effect whilst the other study did not. A summary of *in vivo* studies investigating the tissue response to FLASH-RT compared to CONV-RT, across a range of tissue types, are shown in Tables 1, 2, many of which have demonstrated a reduction in radiation-induced toxicities for FLASH-RT (10–16, 24–27, 34).

**Table 1:** Summary of irradiation parameters and outcomes for *in vivo* studies investigating the FLASH effect in normal tissues (organized in order of model species and targeted tissue, as well as color coded by radiation modality).

***In vivo*** **studies**			**Irradiation delivery technique**			
**Model**	**Assay**	**FLASH dose modification factor** **(Bold if >1)**	**Total dose** **(Gy)**	**Dose rate** **(Gy/s)**	**Pulse rate** **(Hz)**	**Modality of radiation**
Zebrafish embryo (16)	Fish length	**1.2–1.5**	10–12	10^6^-10^7^	Single pulse	Electron
Zebrafish embryo (29)	Fish length, survival, and rate of oedema	1	0–43	100	0.106 ×10^9^	Proton
Whole body irradiation of mice (34)	LD50	**1.1**	8–40	17–83	400	Electron
Thoracic irradiation of mice (10)	TGFβ signaling induction	**1.8**	17	40–60	100–150	Electron
Thoracic irradiation of mice (18)	Number of proliferating cells, DNA damage, expression of inflammatory genes	**>1** **Significant Differences**	17	40–60	100–150	Electron
Abdominal irradiation of mice (33)	Survival	<1 Significant Difference	16	35	Likely 300	Electron
Abdominal irradiation of mice (12)	LD50	**1.2**	22	70–210	100–300	Electron
Abdominal irradiation of mice (17)	Survival, stool formation, regeneration in crypts, apoptosis, and DNA damage in crypt cells	**>1** **Significant Differences**	12–16	216	108	Electron
Whole brain irradiation of mice (25)	Novel object recognition and object location tests	**>1** **Significant Differences**	30	200, 300	108, 180	Electron
Whole brain irradiation of mice (13)	Variety of neurocognitive tests	**>1** **Significant Differences**	10	5.6·10^6^	Single pulse	Electron
Whole brain irradiation of mice (14)	Novel object recognition test	**>1** **Significant Differences**	10	30–5.6·10^6^	100 or single pulse	Electron
Whole brain irradiation of mice (8)	Novel object recognition test	**≥1.4**	10	5.6–7.8·10^6^	single pulse	Electron
Whole brain irradiation of mice (24)	Novel object recognition test	**>1** **Significant Difference**	10	37	1,300	X-ray
Total body and partial body irradiation of mice (32)	TD50	1	3.6–28	37–41	1,388	X-ray
Thoracic irradiation of mice (11)	lung fibrosis, skin dermatitis, and survival	**>1** **Significant Difference**	15, 17.5, 20	40	?	Proton
Irradiation of mouse tail skin (49)	Necrosis ND50	**1.4**	30 and 50	17–170	50	Electron
Irradiation of mouse skin (27)	Early skin reaction score	**1.1–1.6**	50–75	2.5 mean, 3 ×10^4^ in the pulse	23–80	Electron
Irradiation of rat skin (26)	Early skin reaction score	**1.4–1.8**	25–35	67	400	Electron
Irradiation of mini-pig skin (15)	Skin toxicity	**≥1.4**	22–34	300	100	Electron

**Table 2:** Summary of irradiation parameters and outcomes for *in vivo* studies investigating the FLASH effect in tumor tissues (organized in order of model species and targeted tissue, as well as color coded by radiation modality).

***In vivo*** **studies**			**Irradiation delivery technique**			
**Model**	**Assay**	**FLASH dose modification factor** **(Bold if >1)**	**Total dose (Gy)**	**Dose rate (Gy/s)**	**Pulse rate (Hz)**	**Modality of radiation**
Thoracic irradiation of orthotopic engrafted non-small cell lung cancer (Lewis lung carcinoma) in mice (36)	Tumor size and T-cell Infiltration	**>1** **Differences in tumor size (significant) and T-cell infiltration**	18	40	?	Proton
Thoracic irradiation of orthotopic engrafted mouse lung carcinoma TC-1 Luc+ in mice (10)	Survival and tumor Growth Delay	1	15-28	60	100–150	Electron
Abdominal irradiation of mice (17)	Number of tumors, tumor weights	1	12–16	216	108	Electron
Whole brain irradiation of nude mice with orthotopic engrafted H454 murine glioblastoma (8)	Tumor Growth Delay	1	10–25	2.8–5.6·10^6^	Single pulse	Electron
Local irradiation of subcutaneous engrafted Human breast cancer HBCx-12A and head and neck carcinoma HEp-2 in nude mice (10)	Tumor Growth Delay	1	15–25	60	100–150	Electron
Local irradiation of subcutaneous engrafted U87 human glioblastoma in nude mice (8)	Tumor Growth Delay	1	0–35	125–5.6·10^6^	100 or single pulse	Electron
Local irradiation of subcutaneous engrafted U87 human glioblastoma in nude mice (19)	Tumor Growth Delay	1	10–30	125–5.6·10^6^	100 or single pulse	Electron
Local irradiation of subcutaneous engrafted Human hypopharyngeal squamous cell carcinoma ATCC HTB-43 in nude mice (35)	Tumor Growth Delay in irradiated Mice and RBE	1	20	0.008 mean, ≈10^9^ in pulse	< <1	Proton
Treatment of locally advanced squamous cell carcinoma (SCC) in cat patients (15)	Tumor response and survival	1 Similar response as in published studies with CONV-RT	25–41	130–390	100	Electron
Treatment of CD30+ T-cell cutaneous lymphoma T3 N0 M0 B0 in human patient (9)	Tumor response	1 Similar response as previous treatments with CONV-RT	15	167	100	Electron

## Similar Anti-Tumor Response With Flash-Rt As Conv-Rt

In addition to limiting toxicities, there have also been reports of FLASH-RT maintaining the same tumor response as seen following CONV-RT (8, 10, 17, 19, 35). In one such study, breast cancer, and head and neck carcinoma xenografts were established in mice (10). Both tumor models were then exposed to either FLASH-RT or CONV-RT; tumor volume was controlled independent of dose rate in breast, and head and neck xenografts. In the same study, mouse lung carcinoma luciferase-positive (luc+) TC-1 cells were transpleurally injected to generate an orthotopic lung tumor model. Thoracic irradiation of the mice with either CONV-RT or FLASH-RT, and subsequent evaluation of tumor growth using bioluminescence, showed no difference in treatment efficacy (10). Similarly in another study, human glioblastoma (GBM) were engrafted to nude mice and locally irradiated with either FLASH-RT or CONV-RT, resulting in similar tumor growth retardation (19). In the study by Bourhis et al. H454-luc+ murine GBM cells were implanted orthotopically in the striatum of nude mice. This was subsequently followed by whole brain irradiation 3 days post-implantation with either single pulse (1.8 μs) FLASH-RT or CONV-RT (0.1 Gy/s) (8). The mice were irradiated with a 10 Gy single fraction, 3 times 8 Gy, or 5 times 5 Gy, with 24 h in-between fractions. Using bioluminescence to assess the tumor burden, no significant difference could be seen between FLASH-RT and CONV-RT for any of the fractionation schemes (8). In a study by Rama et al. Lewis Lung Carcinoma (LLC) cells were inoculated into the left lung of C57Bl/6J mice (36). Two weeks post-inoculation, the whole lungs of tumor-bearing mice were irradiated with a single fraction dose of 18 Gy, using a clinical pencil beam scanning proton system. One week post treatment, CT-scans were performed to measure tumor size. Tumor size was also measured with a caliper after the mice had been sacrificed 10 days post-treatment. Surprisingly, the tumors of the mice treated with proton FLASH-RT were smaller than the tumors of the mice treated with proton CONV-RT. Moreover, immuno?uorescent staining on harvested tumor sections showed an improved recruitment of T lymphocytes into the tumor microenvironment for tumors treated with FLASH-RT compared to CONV-RT (36). Evidentially in some cases, the anti-tumor response to FLASH-RT might even be better than that of CONV-RT.

## What Factors Influence The Flash Effect?

An important caveat of the pre-clinical studies investigating FLASH-RT is the lack of consistency between variables that could potentially influence the induction of the FLASH effect such as: dose rate, total dose, pulse rate, fractionation, and modality of radiation (Tables 1, 2). The study by Montay-Gruel et al. using a wide range of dose rates has helped to elucidate the extent to which dose rate modulates the FLASH effect (14). As previously described, a neuroprotective FLASH effect was apparent at dose rates ≥ 30 Gy/s with a maximal FLASH effect induced at dose rates ≥ 100 Gy/s. This relationship is important to consider when examining studies such as those by Favaudon et al. (10), and Vozenin et al. (15, 16), which used 40–60 and 300 Gy/s, respectively when administering FLASH-RT. In contrast to previously mentioned studies, a recent interesting study by Venkatesulu et al. showed a higher toxicity for FLASH-RT delivered at 35 Gy/s than for CONV-RT delivered at 0.1 Gy/s (33). This dose rate is probably on the low side for a sparing effect to occur but that does not explain the highly unexpected increased toxicity they found for FLASH-RT in all of their experiments, especially the increased toxicity of a factor 1.3–1.4 for their *in vitro* data. There could be many reasons for these results, e.g., the dose-rate needed for a FLASH sparing effect might not be universal but rather tissue-specific, model and/or assay specific, or there could be dosimetric differences between the two delivery modes/setups, all of which highlights the challenge in performing studies at these dose rates, finding, and exploring a beneficial FLASH effect (33). Furthermore, there is a large degree of variation in the total dose of radiation used in pre-clinical FLASH-RT studies. Compounding this, the majority of studies administer FLASH-RT in single fractions of 10 Gy or more; in many clinical situations, these are currently considered to be extremely large and unattainable fraction doses.

**Figure 1:**
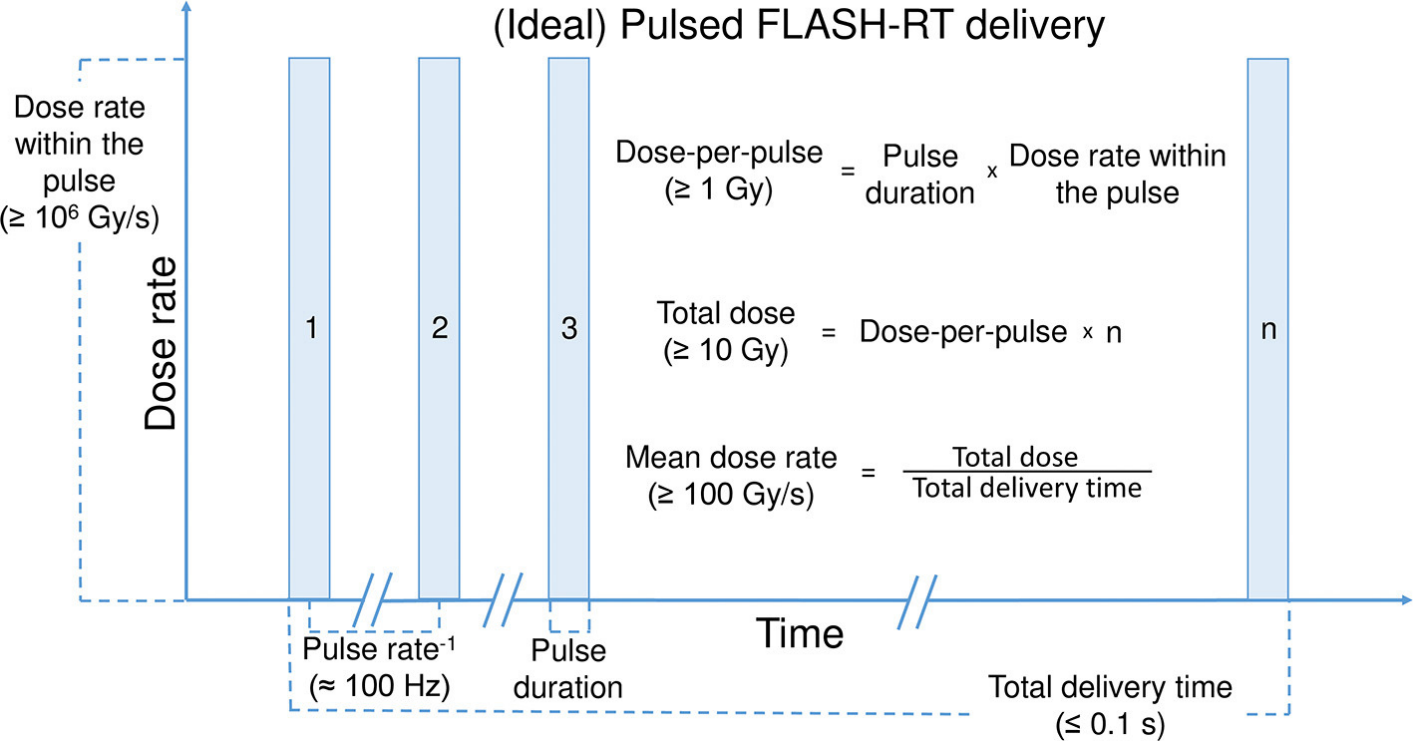
(Ideal) Pulsed FLASH-RT delivery. A schematic view of a pulsed beam delivery, specifying some parameters which seems to be important for inducing the FLASH effect.

The source of radiation must also be considered when evaluating the FLASH effect. The FLASH effect has been predominantly observed following FLASH-RT using dedicated electron linear accelerators as the source of radiation (10, 14, 15, 18, 37). However, recent studies have expanded the FLASH field and include observations of a FLASH effect following proton (11, 23, 36) and X-ray (24) irradiation. Again, it must be noted that there have been a couple of studies that have been unable to induce a FLASH effect using proton and X-ray sources (Table 1). The reason for one X-ray study showing a FLASH effect and one study not showing an effect was discussed above. The proton study compared quasi-continuous proton beam delivery at a CONV-RT dose rate of 5 Gy/min to FLASH-RT of 100 Gy/s, without seeing any toxicity difference for zebrafish embryos (29). A reason for the absent FLASH effect might be the quasi-continuous proton beam delivery with several orders of magnitude lower dose rates within each micro-pulse (≈ 10^3^ Gy/s) than the FLASH electron studies macro-pulses (≈ 10^6^ Gy/s) (29). So, further to mean dose rate, total dose, and the source of radiation, the pulsatile nature of irradiation may also influence the FLASH effect. In order to induce a FLASH effect, it seems that the irradiation beam should ideally be pulsed at a frequency in the order of 100 Hz (Figure 1). Furthermore, within each pulse; irradiation should be delivered at sufficiently high dose-per-pulse, and dose rate within the pulse (≥ 1 Gy and ≥ 10^6^ Gy/s, respectively). Together, resulting in a total treatment delivery time of maximum a few tenths of a second (Table 1). The range of variables and outcomes seen to date warrants further investigation to confirm that these are the key parameters for inducing the FLASH effect (Figure 1).

## Hypotheses To Explain The Flash Effect

### Oxygen Depletion Hypothesis

The biological mechanism responsible for the reduction in normal tissue toxicities following irradiation at FLASH dose rates is not currently understood, yet several non-mutually exclusive hypotheses have been proposed. Some researchers have suggested that the differential response between FLASH-RT and CONV-RT may be due to the radiochemical depletion of oxygen at ultra-high dose rates and subsequent radioresistance conferred to the irradiated tissue (32, 38, 39). It is widely accepted that hypoxic tissues are more radioresistant than well-oxygenated tissues. This is because in the presence of molecular oxygen there is fixation of indirect radiation-induced DNA damage. Indirect damage, the predominant mechanism by which low linear energy transfer (LET) radiation induces DNA damage, occurs when radiation results in the radiolysis of water molecules and the subsequent generation of free radicals. Free radicals are then incorporated into DNA, causing damage—yet this can be easily resolved. However, if a free radical reacts with molecular oxygen, this yields a peroxyl radical. Peroxyl radicals have the potential to induce permanent damage, and are therefore a more efficacious DNA damaging agent. Hence, a lack of oxygen in the immediate environment of a cell limits the extent of radiation-induced DNA damage (40).

When considering the oxygen depletion theory, it is important to note the nature of physiologically relevant oxygen concentrations, or “physoxia” (41). Normal tissues *in vivo* are perfused at much lower oxygen concentrations than *in vitro* cell lines cultured in atmospheric oxygen concentrations. Depending on tissue type, physoxia generally lies between 3.4 and 6.8% oxygen (42). Especially relevant for current treatment with FLASH-RT limited to superficial tissues, physoxia in skin increases with depth from the surface of the skin to the dermis, from around 1.1–4.6% (43). Considering physoxia, and given the critical relationship between oxygen concentration and radiosensitivity radiochemical oxygen depletion has the potential to significantly dampen the radiobiological response.

A relationship between dose rate and oxygen consumption was proposed by Dewey and Boag in 1959 (44). They demonstrated that bacteria irradiated at ultra-high dose rates had greater survival compared to bacteria irradiated at what we now consider to be conventional dose rates. The survival curve generated following ultra-high dose rate irradiation was indicative of bacteria irradiated in a hypoxic environment. The authors hypothesized at the time that this response was a consequence of oxygen depletion following a large dose of radiation in such a short timeframe; the time for which the bacteria were irradiated for was shorter than the time required for oxygen to diffuse and restore the oxygen that had been depleted. Given that molecular oxygen is depleted as it reacts with free radicals generated from the radiolysis of water, irradiation at ultra-high dose rates is able to significantly deplete oxygen before it can replenish. This gives rise to a small window of radiobiological hypoxia.

The oxygen-depletion hypothesis has been strengthened by work demonstrating that as dose rate is increased, cellular survival mimics that of cells irradiated in an increasingly hypoxic environment (45, 46). Furthermore, it was subsequently shown in mammalian cells that the oxygen-dependent fixation of indirect DNA damage could be dampened at ultra-high dose rates (47). Importantly, the total dose at which these cells exhibited a hypoxic-like response was linear with respect to increasing the oxygen concentration in which the cells were cultured. The range of oxygen concentrations used in this study was relatively narrow (0.44–0.7% O_2_) and therefore the phenomenon could have been limited to cells already in hypoxic environments. However, the recent *in vitro* study by Adrian et al. used physiologically relevant oxygen concentrations (1.6–8.3% O_2_) and showed that the sparing effect of FLASH irradiation is dependent on oxygen concentration (48). An *in vivo* mouse model has also shown that irradiation of mouse tails at ultra-high dose rates induced radioresistance indicative of oxygen depletion (49).

**Figure 2:**
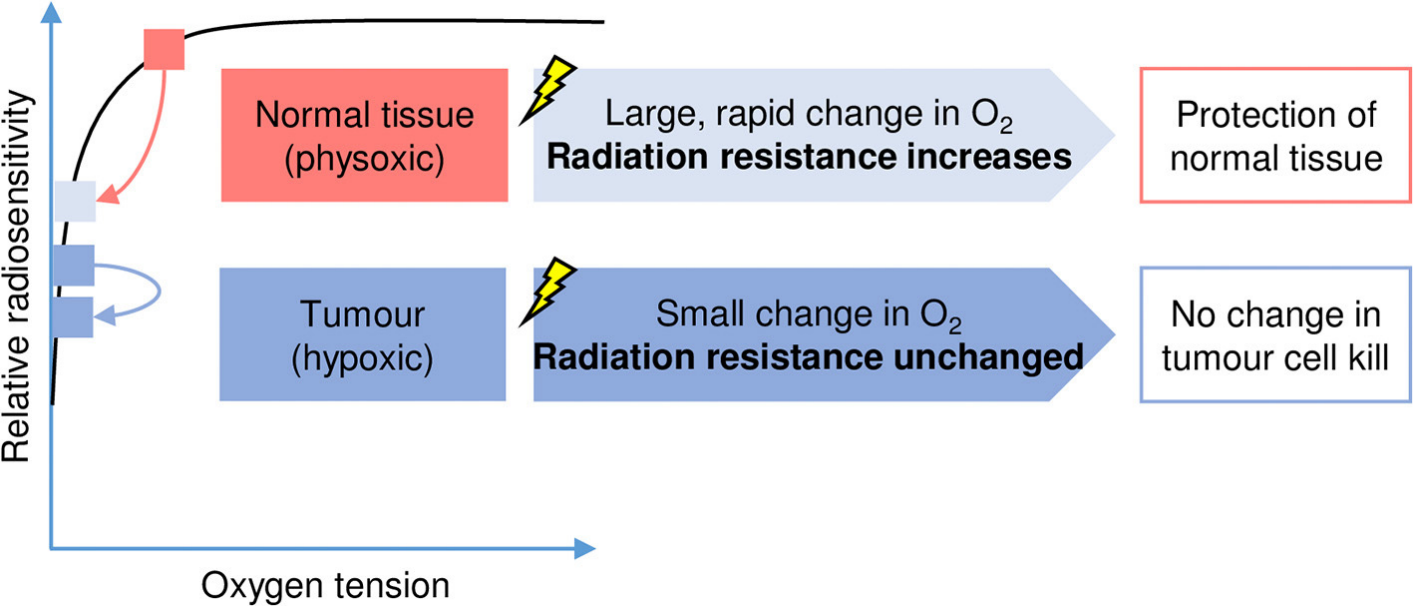
The oxygen depletion hypothesis. The relationship between oxygen tension (horizontal axis) and radiation sensitivity (vertical axis) is shown schematically and has been widely reported (40, 41). In response to FLASH-RT, the physiological level of oxygen (physoxic) found in normal tissues decreases rapidly (pink arrow) and has an important impact on radiation sensitivity. This temporary or transient hypoxia protects the normal tissues as radiation resistance increases. In contrast, oxygen levels are low (hypoxic) in tumor tissues and consequently FLASH-RT has less of an impact on radiation sensitivity.

Together, these data suggest that the irradiation of tissues with FLASH-RT results in radiochemical oxygen depletion, giving rise to an extremely acute period of hypoxia within the irradiated tissue and consequently a transient radioresistance (Figure 2). This phenomenon is not seen following irradiation with CONV-RT as radiation is delivered with much smaller pulses and over a longer timeframe. Hence during CONV-RT, oxygen depletion is limited, and there is sufficient time for oxygen to diffuse into the irradiated region to replace oxygen that has been lost. Therefore, oxygen concentration within the irradiated tissue is maintained.

There is growing interest surrounding other oxygen-based radicals as a potential mechanism bridging the local oxygen depletion observed following irradiation at ultra-high dose rates, and reduced toxicities to normal tissue. A recent study proposes that oxygen depletion at ultra-high dose rates promotes the protection of normal tissue by limiting the production of reactive oxygen species (ROS) (13). This study repeated previous work, demonstrating that whole brain irradiation of C57Bl6/J mice with FLASH-RT did not induce cognitive impairments at dose rates exceeding 100 Gy/s compared to non-irradiated controls. Moreover, in support of a critical role for oxygen in the FLASH effect, increasing the local oxygen concentration in mice brains through carbogen breathing reversed the cognitive protection conferred by FLASH-RT. Furthermore, zebrafish embryos were subjected to either FLASH-RT or CONV-RT in the presence or absence of two well-documented ROS scavengers: *N*-acetyl-cysteine (NAC), and amifostine (13). Giving weight to the involvement of ROS in the FLASH effect, zebrafish embryos exposed to FLASH-RT in combination with a ROS scavenger had no effect on zebrafish length 5 days post-irradiation. However, zebrafish embryos exposed to CONV-RT alone were significantly shorter than those exposed to CONV-RT in combination with a ROS scavenger (13). This provides crude but encouraging evidence suggesting that toxicities arising from CONV-RT are in part due to the generation of ROS, and that the generation of these species is reduced following FLASH-RT. The largest limitation of this study is that there are no direct measurements of ROS in a physiological context. Instead, water containing 4% aqueous oxygen was irradiated at either ultra-high or conventional dose rates; conventional dose rates generated significantly greater ROS than ultra-high dose rates (13). Despite this short fall, the interesting findings detailed upon irradiation in combination with antioxidants merits further exploration into the role of ROS for the FLASH effect.

The oxygen depletion hypothesis seems to explain the reduced toxicity of FLASH-RT to normal tissue. However, it does not easily explain how FLASH-RT can maintain tumor response relative to CONV-RT. Although tumors are more hypoxic compared to their normal tissue counterparts, most are not completely anoxic (42). Therefore, following FLASH-RT, there will also be radiochemical depletion of oxygen within the tumor, hence it would be expected that this would confer radioresistance to the tumor. In contrast to experimental data (8, 10, 19), one would subsequently expect to observe reduced tumor control following FLASH-RT relative to CONV-RT. Though, for highly hypoxic tumor models the reduced tumor control would be expected to be minimal (Figure 2). A possible explanation for the maintained tumor control is proposed in a recent paper by Spitz et al. They hypothesized that higher levels of redox-active iron (labile iron) in tumor compared to normal tissue and differences in oxidative metabolism between normal and tumor tissues, with the more rapid removal and decay of the organic hydroperoxides and free radicals derived from peroxidation chain reactions in normal tissue, defines the beneficial therapeutic index of the FLASH effect (50). Interestingly, a recent computational model of oxygen depletion induced by FLASH-RT concluded that radiochemical oxygen depletion at an expected rate of 0.42 mmHg/Gy would be sufficient to confer radioresistance (51). However, this conclusion was predicated on the basis that radioresistance would only be conferred to already hypoxic tissues. To explore this, it would be interesting to compare the DNA repair proficiency of normal tissue relative to tumor tissue; perhaps radioresistance induced in tumor tissue by oxygen depletion is compensated for by a lower ability of DNA repair compared to normal tissue. Regions of hypoxia occur in the majority of solid tumors as opposed to the physoxia found in the surrounding normal tissue. This may well be relevant to the relative repair of DNA damage induced by FLASH-RT as exposure to hypoxia has also been described to lead to the repression of the DNA repair pathways including homologous recombination (HR), non-homologous end joining (NHEJ), and base excision repair (BER) (52, 53). To test this hypothesis, the rate of DNA repair, assayed for example by determining the appearance and resolution of 53BP1 foci, should be measured in both normal and tumor cells after exposure to FLASH-RT.

The vast majority of data pertaining to the oxygen depletion theory has been extrapolated from cell survival responses following irradiation at different dose rates (44–47, 49, 54). Therefore, there must be more direct measurements of any potential oxygen flux in tissues following irradiation at ultra-high dose rates. However, given the supposed brevity of any hypoxia induced by FLASH-RT, this is extremely difficult; it has been inferred that reoxygenation by diffusion of a tissue following FLASH-RT occurs after just 10^−3^ s (54). Hypoxia for such a brief moment can certainly not be detected by measuring markers of a hypoxia-mediated transcriptional response, which would be observed following a longer period of hypoxia (41). However, it is unknown whether a chemical marker of hypoxia, such as pimonidazole (55) is sufficiently sensitive to detect such an acute period of hypoxia.

### Immune Hypothesis

A modified immune response following FLASH-RT relative to CONV-RT has also been proposed as a potential mechanism for the FLASH effect (9, 38). The fractionated RT regimes commonly used in CONV-RT, result in the irradiation of a greater proportion of circulating lymphocytes compared to total dose delivered in a single fraction (56). Following a standard regime of thirty fractions of 2 Gy, 98.8% of the blood pool has been exposed to more than 0.5 Gy. Additionally, it has been reported that the induction of chromosomal aberrations in the circulating blood pool is dependent on the total volume of the blood pool irradiated (57). Therefore, in accordance with the short irradiation time, characteristic of FLASH-RT, it would follow that fewer lymphocytes would be irradiated and subsequently reduced induction of chromosomal aberrations (9, 38, 56). However, FLASH-RT would expose lymphocytes to a greater dose of radiation, albeit much fewer of them, in comparison to CONV-RT. If a modified immune response contributes to the FLASH effect, one would expect a fractionated FLASH-RT regime to, at least in part, reduce any protection conferred by the FLASH effect.

This hypothesis has been strengthened recently by a study that carried out genome-wide microarray analysis on mice following FLASH-RT and CONV-RT (11). This study reported that immune system wide activation and maturation was dampened in mice following FLASH-RT relative to CONV-RT. Also as mentioned above, the study by Rama et al. showed an improved recruitment of T lymphocytes into the tumor microenvironment for tumors treated with FLASH-RT compared to CONV-RT, which gives merit to this hypothesis (36). In several studies, immunocompromised animals were used to compare treatment efficacy of FLASH-RT and CONV-RT with no observed difference in tumor response (Table 2), which could be interpreted to further strengthen the hypothesis (7, 8, 10, 35). It is worth noting however, that any evidence linking an immune role to the FLASH effect is correlative rather than causative; it is unclear whether any differential immune response following irradiation at ultra-high dose rates contributes to the FLASH effect, or is a consequence of it. Additionally, since the FLASH effect has been observed *in vitro* in bacterial and cell culture models, which are devoid of a functioning immune system, any immunological component is likely to be responsible for only part of the underlying mechanism. More studies are needed to clarify if the immune response or other biological responses like DNA damage response or inflammation is different following FLASH-RT compared to CONV-RT, and if they are part of the underlying mechanism resulting in the FLASH effect.

## Clinical Applications Of Flash-Rt

The obvious endpoint of investigation into the FLASH effect is the translation of FLASH-RT to the clinic. FLASH-RT could be translated to the clinic to serve two general purposes. Firstly, the FLASH effect could be exploited to allow for escalation of total dose in the treatment of radioresistant tumors that are currently associated with poorer patient outcomes (8). In this case, it is hypothesized that a greater dose of radiation could be delivered to the tumor without inducing as severe toxicities to the normal surrounding tissue as would be expected following CONV-RT. Secondly, FLASH-RT could be used in situations in which RT confers good levels of tumor control but is associated with severe normal tissue toxicity—the same total dose would be administered, but hypothetically FLASH-RT would induce less severe toxicities compared to CONV-RT.

Despite these exciting potential applications of FLASH-RT, the extent to which it is clinically viable in practice is questionable. As reviewed above, there are some inconsistencies in the results from the pre-clinical studies. Furthermore, a proportion of these studies are designed with significant limitations, such as using a single subject and a lack of controls irradiated at conventional dose rates (15). Moreover, the results emerging from pre-clinical studies put into question the suitability of FLASH-RT in many clinical situations. Independent studies that have successfully observed a FLASH effect report a dose-modifying factor of about 20–40% in favor of FLASH-RT relative to CONV-RT (Table 1). However, these same studies only report a FLASH effect at total doses of 10 Gy or more. This point is particularly well-illustrated in the recent study by Vozenin et al. (16). In a zebrafish model, whereby zebrafish embryos were irradiated with FLASH-RT or CONV-RT at doses ranging from 5 to 12 Gy, increasing in 1 Gy increments, zebrafish length was recorded 5 days post-irradiation as a measure of radiation-induced toxicity. A significant difference in morphology between those irradiated with FLASH-RT or CONV-RT was only apparent at doses ≥ 10 Gy. Even when accounting for the dose modifying factor of FLASH-RT, an equivalent dose per fraction of 6–8 Gy given by CONV-RT may still be considered as too large a dose in various clinical scenarios (58–60), such as in the treatment of larger, locally advanced tumors. A previous phase I dose escalation study in locally advanced non-small cell lung cancer (NSCLC) utilized hypofractionated treatment with doses per fraction well-below those required for a FLASH effect (58). Six patients developed late onset, grade 4–5 toxicities that were attributed to damage to the proximal bronchial tree, ergo highlighting the need for caution when employing hypofractionated regimes. Hypofractionation is nevertheless getting more widely used in the clinic for a variety of treatments sites (59, 61–64), and could be proven even more useful together with FLASH-RT and its (potentially) lower level of normal tissue toxicity.

One of the most interesting advancements in the FLASH field is the first human patient treated with FLASH-RT (9). A 75-year-old male presenting with multiresistant CD30+ T cell cutaneous lymphoma was offered the opportunity to be first human subject of FLASH-RT. A 35 mm lesion was exposed to a dose rate exceeding 10^6^ Gy/s in each of ten discreet 1 μs pulses to a total dose of 15 Gy. This equates to a mean dose rate of 167 Gy/s, and 1.5 Gy per pulse. Following treatment, shrinkage of the lesion was observed 10 days post-irradiation culminating in a complete tumor response 36 days post-irradiation which was maintained for the following 5 months. From the point at which the lesion initially began to shrink, the patient presented with redness and mild (grade 1) oedema and epithelitis around the site of irradiation. This was starkly different to the patient's other lesions treated with CONV-RT that resulted in high-grade acute reactions to the surrounding skin that took ~3–4 months to heal (9). Despite the promising outcome for this patient, this should not be considered evidence confirming that FLASH-RT can be successfully translated to the clinic. This study was performed in a single patient that only allowed for limited comparison of the differential response between FLASH-RT and CONV-RT. An appropriately powered, randomized controlled trial with FLASH-RT and CONV-RT arms would be required to definitively show whether FLASH-RT is associated with superior clinical outcomes. At the very least, a positive phase II, single-arm study of FLASH-RT in a sample of participants truly representative of real-world patients is required before the routine adoption of FLASH-RT can be seriously entertained. If 4.5–20 MeV electron beams are to be used for the clinical trials, they would be limited to treating surficial tumors or treating tumors with intra-operative radiation therapy (IORT). Currently, FLASH-RT clinical trials on deep-seated tumors can only be performed with proton beams (Table 3). However, to treat tumors with a proton beam in a clinical trial, the beam needs to be scattered or scanned to cover the target volume which reduces the average dose rate (65). So before performing clinical trial, pre-clinical studies are needed to ensure that the FLASH effect is not lost due to either the increased LET in the Bragg peak or to the required scattering/scanning of the beam.

**Table 3:** Some relevant advantages and disadvantages of current and prospective FLASH radiotherapy sources (color coded by radiation modality).

**Radiation source**	**Modality of radiation**	**Advantages (+)**	**Disadvantages (–)**	**Currently available for FLASH-RT clinical studies, with which main limitations?**
Conventional electron linear accelerator (10, 14, 66, 67)	1–25 MeV Electrons	Inexpensive. Minor beam size limitation.	Poor depth penetration. Wide penumbra.	Yes, Limited to treating superficial tumors.
Very High Energy Electron linear accelerator (68, 69) or Laser plasma accelerators (70, 71)	100–250 MeV Electrons	Good depth penetration. Electromagnetic steering and focusing. Not sensitive to tissue heterogeneity.	Low pulse rate (1–10 Hz) for Laser plasma accelerators. Limited beam size.	No
Laser plasma accelerators (75)	1–45 MeV Protons	Compact design possible. Electromagnetic steering possible.	Poor depth penetration. Low pulse rate (1–10 Hz). Very sensitive to tissue. heterogeneity. Higher LET in Bragg peak. Beam contamination. Stability issues. Limited beam size.	No
Cyclotrons, synchrotrons or Synchrocyclotron (11, 76)	100–250 MeV Protons	Good depth penetration. Electromagnetic steering possible. Limited dose-bath. Electromagnetic steering.	Large expensive sources. Sensitive to tissue heterogeneity. Higher LET in Bragg peak. Beam scanning or scattering required to cover target volumes	Yes, FLASH effect might be lost with beam scanning and/or higher LET.
X-ray tube (72)	50–250 keV X-rays	Inexpensive. Compact design.	Very limited depth penetration. Limited beam size. High entrance dose.	Yes, Limited to treating small and very superficial tumors.
Synchrotron (24, 32)	50–600 keV X-rays	Microbeam Radiation Therapy possible.	Very large. Very expensive. Limited depth penetration. Very limited availability. Limited beam size requires scanning of sample/target.	Yes, Very limited availability.
Electron linear accelerator with high density target (20)	6–10 MV X-rays	Good depth penetration. Narrow penumbra. Minor beam size limitation.	Multiple beam angles required.	No

As previously mentioned, most studies showing a FLASH effect has dedicated electron linear accelerators as the source of radiation (9, 10, 14, 15, 18, 37). Recent studies have shown that clinical linear accelerators can be modified to deliver FLASH-RT with electrons, largely increasing the potential availability of FLASH-RT devices and facilitating the translation to clinical trials (66, 67). However, an obvious limitation is the depth penetration with 4.5–20 MeV electron beams, only reaching to a few cm depths in tissue (Table 3). Consequently, other treatment devices/techniques are needed for FLASH-RT to be clinically useful for more than superficial treatments with external beam RT or IORT. A solution to the limited depth penetration would be to use electron beams of higher energy, so called Very High Energy Electron (VHEE) beams, with beam energies of 100–250 MeV. Such beams have good depth penetration, sharp beam penumbra, and are less sensitive to tissue heterogeneity than conventional X-ray beams (68, 69). Also, using electromagnets, the beam can *in theory* be focused to the tumor volume, resulting in dose-to-target conformity with a single beam comparable to that of modern X-ray treatment techniques, e.g., intensity-modulated radiation therapy (IMRT) and volumetric modulated arc therapy (VMAT). A single beam delivery might prove essential for retaining the FLASH effect in clinical trials. Unfortunately, these beams are currently limited to research accelerators which are either rather large (linear accelerator) or suffers from a low pulse rate, a small beam size, and stability issues (laser-based accelerators) (68–71). A recent paper showed (using a 160 kV X-ray beam) that conventional X-ray tubes could potentially be used for FLASH-RT studies (72). This is interesting as such systems are small, relatively inexpensive and clinically available (Table 3). Similar however to the electron linear accelerators, the depth penetration is a limiting factor making it useful only down to a few mm depth in tissue, an additional limitation is the beam size of only a few cm. Synchrotron sources has similar beam energies as X-ray tubes but has the added advantage of the possibility of using spatially fractionated ultra-high dose rate microbeam radiation therapy (MRT). MRT is characterized by arrays of quasi-parallel micro-planar beams with a width of 25–100 μm, typically separated by 100–400 μm (32). Since its invention in 1992, numerous pre-clinical studies have shown extraordinary tolerance of normal organs and blood vessels exposed to fractionated radiation doses in excess of 100 Gy in-beam (peak) doses, with dose rates exceeding several hundred Gy/s. The combined effect of spatially fractionated microbeams and FLASH dose rates have been shown in small animal models to achieve therapeutic ratios that clearly exceed those obtained by conventional X-ray with a homogeneous dose distribution and CONV-RT dose rates, in a range of malignancies, including gliomas, gliosarcomas, human squamous cell carcinomas, and glioblastomas (73). The disadvantage of this technique is the requirement of synchrotrons, which are very large, expensive, and therefore of limited availability. A platform that might solve both the size and stability issue of VHEE beams and also allow for the production of 6–10 MV FLASH X-ray beams, is PHASER (Pluridirectional High-energy Agile Scanning Electronic Radiotherapy). The PHASER concept has been presented by Maxim et al. and might be an ideal way for introducing FLASH into the clinic (20). Included in the concept is a novel and quick image-guided technique. New or highly adapted image-guidance techniques are needed for the clinical treatment of deep-seated tumors with FLASH-RT, regardless of radiation modality. The PHASER is reliant on technical advances and novel innovations in linear accelerator technology, radiofrequency science and medical physics, which in turn requires time and funding for research and development. Therefore, it is still under development (Table 3). Alternative concepts of producing 6–10 MV FLASH X-ray beams would be to use multiple synchronized linear accelerators or a powerful recirculating accelerator (74). Albeit large and expensive, a clinically available system for treating deep-seated tumors with FLASH-RT is with proton beams (75, 76). Clinical proton beams have good depth penetration, are often electromagnetically steered, and can produce conformal dose distributions with a single to a few beams (65). There have been studies (published and unpublished) with mixed reports on a FLASH effect with protons but significant resources have now been put into research on proton FLASH-RT by the principal vendors for proton RT devices, which should expedite the translation of proton FLASH-RT into clinical trials (77–79).

## Conclusion

The FLASH effect is an extremely interesting radiobiological phenomenon that confers some degree of protection compared to CONV-RT. The FLASH effect has now been observed across a range of animal models, and more recently has been suggested in a human patient for the first time. Of equal importance, limited data would suggest that FLASH-RT maintains a similar tumor response to CONV-RT. Together, this raises the prospect that FLASH-RT will allow patients to receive a greater total dose of radiation prior to the induction of unacceptable toxicities that currently limit RT regimes.

There has been much speculation regarding the biological mechanism(s) underpinning the FLASH effect. It is well-established that irradiation results in the radiochemical depletion of oxygen; this is particularly prevalent at ultra-high dose rates. From the data currently available, we can safely conclude that oxygen depletion contributes, at least in part, to the FLASH effect. However, the extent of its contribution remains unknown and therefore warrants further investigation. Aside from oxygen depletion, an immune modulatory role has been broadly implicated in the FLASH effect, yet evidence to support this is currently sparse and preliminary. Likewise, any potential immune-mediated contribution to the FLASH effect requires much greater exploration.

Aside from mechanistic insights, the overarching question remains of the translational potential of FLASH-RT to clinical environments. Despite independent studies concluding that FLASH-RT confers a dose modifying factor of 20–40%, the repeated finding that the FLASH effect is only evident at total doses of 10 Gy or more means that FLASH-RT would not be suitable in many clinical cases. As a result of further investigation into the biological basis of the FLASH effect, it may eventually be possible to generate a FLASH effect at smaller doses, therefore further increasing the clinical potential of FLASH-RT. Another limiting factor in translating FLASH-RT to the clinic is the availability of radiation sources, capable of producing beams suitable for treatment of deep-seated as well as superficial tumors with ultra-high dose rates. In summary, with shorter treatment times and lower levels of toxicity, FLASH-RT may 1 day have the potential to be a paradigm shift in the field of RT. For this to be the case, however, there is a real need to identify the mechanism(s) behind the FLASH effect. The currently available data more than justifies this further investigation.

## Author Contributions

JW and KP wrote the article. EH and GH contributed ideas to, read, and edited the article.

### Conflict of Interest

The authors declare that the research was conducted in the absence of any commercial or financial relationships that could be construed as a potential conflict of interest. The reviewer M-CV declared a past supervisory role with one of the authors KP to the handling editor.
