# Corrigendum: Ultra-High Dose Rate (FLASH) Radiotherapy: Silver Bullet or Fool's Gold?

**DOI:** 10.3389/fonc.2020.00210

**Published:** 2020-02-25

**Authors:** Joseph D. Wilson, Ester M. Hammond, Geoff S. Higgins, Kristoffer Petersson

**Affiliations:** ^1^Department of Oncology, The Oxford Institute for Radiation Oncology, University of Oxford, Oxford, United Kingdom; ^2^Radiation Physics, Department of Haematology, Oncology and Radiation Physics, Skåne University Hospital, Lund, Sweden

**Keywords:** FLASH, radiotherapy, hypoxia, normal tissue, immune

In the original article, there was an error. Hydroxyl radicals are mentioned on four occasions in the text. It should be free radicals and not hydroxyl.

A correction has been made to the Hypotheses To Explain The Flash Effect section, subsection Oxygen Depletion Hypothesis, paragraphs 1 and 3.

“The biological mechanism responsible for the reduction in normal tissue toxicities following irradiation at FLASH dose rates is not currently understood, yet several non-mutually exclusive hypotheses have been proposed. Some researchers have suggested that the differential response between FLASH-RT and CONV-RT may be due to the radiochemical depletion of oxygen at ultra-high dose rates and subsequent radioresistance conferred to the irradiated tissue (32, 38, 39). It is widely accepted that hypoxic tissues are more radioresistant than well-oxygenated tissues. This is because in the presence of molecular oxygen there is fixation of indirect radiation-induced DNA damage. Indirect damage, the predominant mechanism by which low linear energy transfer (LET) radiation induces DNA damage, occurs when radiation results in the radiolysis of water molecules and the subsequent generation of free radicals. Free radicals are then incorporated into DNA, causing damage—yet this can be easily resolved. However, if a free radical reacts with molecular oxygen, this yields a peroxyl radical. Peroxyl radicals have the potential to induce permanent damage, and are therefore a more efficacious DNA damaging agent. Hence, a lack of oxygen in the immediate environment of a cell limits the extent of radiation-induced DNA damage (40).

A relationship between dose rate and oxygen consumption was proposed by Dewey and Boag in 1959 (44). They demonstrated that bacteria irradiated at ultra-high dose rates had greater survival compared to bacteria irradiated at what we now consider to be conventional dose rates. The survival curve generated following ultra-high dose rate irradiation was indicative of bacteria irradiated in a hypoxic environment. The authors hypothesized at the time that this response was a consequence of oxygen depletion following a large dose of radiation in such a short timeframe; the time for which the bacteria were irradiated for was shorter than the time required for oxygen to diffuse and restore the oxygen that had been depleted. Given that molecular oxygen is depleted as it reacts with free radicals generated from the radiolysis of water, irradiation at ultra-high dose rates is able to significantly deplete oxygen before it can replenish. This gives rise to a small window of radiobiological hypoxia”.

The authors apologize for this error and state that this does not change the scientific conclusions of the article in any way. The original article has been updated.

